# ﻿The scorpions of the Estación de Biología Chamela, Jalisco, Mexico with the description of a new species of *Mesomexovis* (Scorpiones, Vaejovidae) and an identification key

**DOI:** 10.3897/zookeys.1243.146978

**Published:** 2025-06-27

**Authors:** André Felipe de Araujo Lira, Edmundo González-Santillán

**Affiliations:** 1 Colección Nacional de Arácnidos, Departamento de Zoología, Instituto de Biología, Universidad Nacional Autónoma de México, 3er. Circuito Exterior S/N, Ciudad Universitaria, Alcaldía Coyoacán, C.P. 04510, Mexico City, Mexico Universidad Nacional Autónoma de México Mexico City Mexico

**Keywords:** Biodiversity, Syntropinae, taxonomy, tropical deciduous forest, Wallacean shortfall

## Abstract

*Mesomexovis* Gonzalez-Santillan & Prendini, 2013 is a scorpion genus of the Vaejovidae family that comprises seven species, all endemic to Mexico. The present study describes a new species from Estación de Biología Chamela, Jalisco, related to *M.occidentalis* (Hoffmann, 1931), *M.atenango* (Francke & Gonzalez-Santillan, 2006), and *M.subcristatus* (Pocock, 1898). *Mesomexoviscaxcan***sp. nov.** differs from these species in several respects. Firstly, the carinae of the pedipalp chelae are vestigial. Secondly, the ventral lateral carinae of metasomal segments I–IV are granular, and the ventral submedian carinae of segments I–IV are costate to granular. A microstructural separation between the subex and the basal carina of the capsular area of the hemispermatophore is described for the first time. *Mesomexoviscaxcan***sp. nov.** represents the eighth species of the genus and the fifth reported in the Estación de Biología Chamela. The other scorpions identified in this location are *Centruroideschamela* Ponce-Saavedra & Francke, 2011, *C.elegans* (Thorell, 1876) (Buthidae), *Konetontlichamelaensis* (Williams, 1986), and *Thorelliusintrepidus* (Thorell, 1876) (Vaejovidae).

## ﻿Introduction

Mexico possesses one of the most diverse scorpion faunas in the world with 38 genera, 313 species, and four subspecies, distributed across nine families (Ponce-Saavedra et al. 2023; Rein 2023; Contreras-Félix and Navarrete-Heredia 2024). There are also several regions in Mexico that harbor an impressive number of syntopic scorpion species; for instance, 14 species have been documented in Baja California (Williams 1980; Jiménez-Jiménez and Palacios-Cardiel 2010). The majority of Mexican scorpion diversity, approximately 89%, can be found in the families Vaejovidae (53%), Diplocentridae (19%), and Buthidae (17%) (Ponce-Saavedra et al. 2023). Although the Mexican scorpion fauna has been studied since the beginning of 19^th^ century (e.g., Pocock 1902), recent years have seen the description of many new species (Chávez-Samayoa et al. 2022; Ponce-Saavedra et al. 2022; Teruel et al. 2023; Villa-Corella et al. 2023; Contreras-Félix and Navarrete-Heredia 2024), indicating that the scorpion diversity remains underestimated. Furthermore, a significant Wallacean shortfall remains in our understanding of Mexico’s scorpion fauna, with several areas yet to be accessed or are poorly sampled (Santibáñez-López et al. 2016). As these authors point out, incomplete sampling may lead to a scarcity of information about species distribution.

Surveying local species diversity offers valuable insights for policymakers to protect the ecosystems (Pascual et al. 2023; Golini et al. 2024). Surprisingly, this knowledge is often overlooked in arthropods, favoring charismatic taxa such as mammals and birds, even within protected areas (Chowdhury et al. 2023). Arthropods, including arachnids, represent most of the hidden or unseen biodiversity in protected areas and they are rarely considered in conservation decisions (Delso et al. 2021). Thus, it is crucial to provide information about species diversity, especially in protected areas.

**Figure 1.**
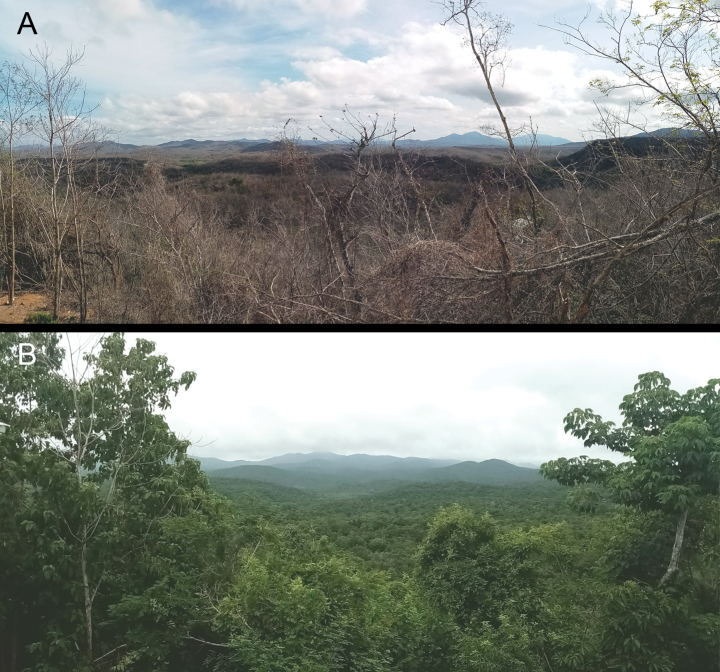
Panoramic view of the tropical deciduous forest at Estación de Biología Chamela **A** dry season **B** rainy season.

The Estación de Biología Chamela (EBC) lies within the Chamela-Cuixmala Biosphere Reserve, one of the largest areas devoted to the preservation and study of tropical dry forest biodiversity on the central Pacific coast (Castillo et al. 2021). One particular aspect of the climatic regimen is a marked seasonality (Fig. 1). Although yearly, four stages were recognized, the rain is restricted to five months and there is a rather unpredictable rainy pattern with total precipitation ranging from 780 mm up to 1390 mm (García-Oliva et al. 2002).

Williams (1986) documented for the first time, four scorpion species for the EBC, among them *Vaejovisoccidentalis* Hoffmann, 1931, now *Mesomexovisoccidentalis*. However, in the redescription of that species Sissom (1989) claimed:

Through the courtesy of Dr. Stanley WILLIAMS, I was able to examine specimens from Chamula [= Chamela], Jalisco which were recently referred to *V.occidentalis* (WILLIAMS, 1986). These specimens represent an undescribed species that is closely related to *V.occidentalis*; males of the two species exhibit considerable differences in body size and morphometrics.

Subsequently, González-Santillán (2004) conducted a comprehensive review of scorpion species richness in the EBC with field collections and specimens deposited in the Colección Nacional de Arácnidos (CNAN), at the Universidad Nacional Autónoma de México (UNAM). This revision revealed the presence of five species, including two undescribed: *Centruroideselegans* (Thorell, 1877), *Centruroides* sp., *Vaejovischamelaensis* Williams, 1986, *Vaejovis* sp. (= *Mesomexovis* sp.), and *V.intrepidus* Thorell, 1876 (= *Thorelliusintrepidus*).

The undescribed species of *Centruroides* was formally described as *Centruroideschamela* Ponce-Saavedra & Francke, 2011. On the other hand, *Vaejovis* sp. was transferred and grouped with seven species into the newly described genus *Mesomexovis* (González-Santillán & Prendini, 2013). These seven species share common morphological characteristics, including a high count of macrosetae on the dorsolateral carinae of metasomal segments III–V and eight retroventral macrosetae on the basitarsus of leg III (González-Santillán and Prendini 2013). In light of the absence of a formal description of the *Mesomexovis* species found at the EBC and a synthesis of the scorpion fauna of the station, this study aims to provide the description of this scorpion species and to offer an updated identification key to families, genus, and species.

## ﻿Materials and methods

Scorpion specimens deposited in the Colección Nacional de Arácnidos (**CNAN**), Instituto de Biología, Universidad Nacional Autónoma de México (IBUNAM) formed the basis of our taxonomic summary. Nomenclature and homology follow González-Santillán and Prendini (2013), except for lateral ocelli patterns (Loria and Prendini 2014), and body measurements (Sissom et al. 1990). Hemispermatophore nomenclature follows Monod et al. (2017) and González-Santillán and Prendini (2013). We obtained standard measurements (mm) with a Nikon SMZ1270 dissecting microscope. Microphotographs were obtained with a digital camera E3ISPM mounted on a stereo microscope Zeiss Discovery.V8, and images manipulated with the software Rising View and edited with Photoshop CS5.1. Morphometric ratios represent the mean of 11 males and 11 females (♂/♀) from the type series, single numbers indicate no sexual morphometric dimorphism.

## ﻿Results

### ﻿Taxonomy

**Family Buthidae C.L. Koch, 1837**

**Subfamily Centruroidinae Kraus, 1955**

#### ﻿ Centruroides chamela

Taxon classificationAnimaliaScorpionesButhidae

Ponce-Saavedra & Francke, 2011

AC3931F0-B6C9-53F2-89ED-6618C2D82D0E

Fig. 2

##### Material examined.

***Holotype*.** México • ♂; Jalisco, Municipio la Huerta: Estación de Biología, IBUNAM; Chamela-Cuixmala, 19°29.88'N, 105°02.61'W, 97 m, 31.VIII.2007, O. Francke, A. Valdez, H. Montaño, A. Ballesteros, and C. Santibáñez leg, collected on the ground; CNAN-T0689. ***Paratypes*.** Same locality • 1 ♂ (CNAN-SC01506), 2.IV.1989, E. Ramírez leg; • 2 ♀, collected inside bromeliads (CNAN-SC01506), collected inside bromeliads; E. Ramírez leg; • 2 ♂, 2 ♀, collected inside bromeliads (AMNH); 2-IV-1989, E. Ramírez leg; • 2 ♂, 2 ♀, collected inside bromeliads (INDRE); 2.IV.1989, E. Ramírez leg; • 1 ♀, collected inside bromeliads (INDRE) 2-IV-1989, E. Ramírez leg, • 1 ♀ (INDRE); • 1 ♂, 1 ♀, collected on the ground (FBUMSNH); • 1 ♂, camino a Chachalacas, 19°29.620'N. 105°02.749'W (FBUMSNH).

**Figure 2.**
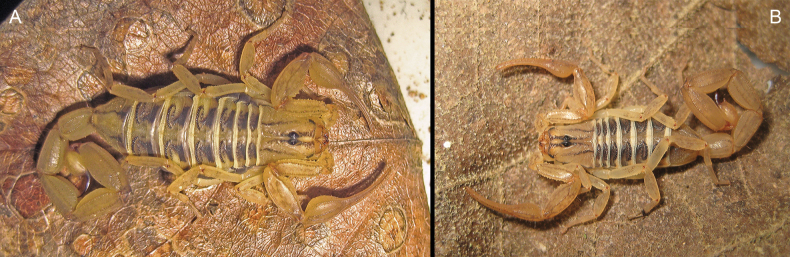
*Centruroideschamela* Ponce-Saavedra & Francke, 2011. Live habitus of adult specimens from EBC**A** female **B** male.

##### Distribution.

This species is endemic to the Pacific lowlands of Jalisco, most likely mirroring the distribution of *C.elegans*.

##### Remarks.

This is the only arboreal member of the striped species group of the genus *Centruroides* (González-Santillán 2004). All other Mexican species of arboreal species are found in the *thorelli* species group, recently revised by Goodman et al. (2021). The authors indicated that the distribution of *Centruroideschanae* Goodman, Prendini, Francke & Esposito, 2021, the only species of the *thorelli* group distributed in the Pacific coast of Mexico, reaches the limits of the littorals of Colima and Michoacán. We infer that *C.chamela* substitutes *C.chanae* ecologically in the littorals of Jalisco. Besides, there are no records of any species resembling species of the *thorelli* group, and our recent fieldwork in the EBC produced only the two species cited here. We tested the potency of the venom of this species and are preparing a report on it; our preliminary results indicate that the potency is comparable to that of *C.elegans*.

#### ﻿ Centruroides elegans

Taxon classificationAnimaliaScorpionesButhidae

(Thorell, 1876)

259AFB4D-A796-51BE-A8A9-C00F991910D6

Fig. 3

##### Type material.

***Holotype*.** Mexico • deposited at the Naturhistoriska Museet, Goteborg, Sweden (not examined).

##### Other material.

Mexico • Jalisco, Municipio la Huerta: Estación de Biología Chamela, 11.5.1989, 19°29.91'N, 105°2.67'W, 80 m, G. Ortega leg (CNAN-SC259), 1 ♂, 2 ♀; • 23.X.2001, 80 m, E. González-Santillán leg (CNAN-SC279), 1 ♂, 2 ♀; • 15.IX.1977, 80 m, N. Pérez (CNAN-SC260), 22 ♂, 4 ♀; • 3.VI.1975, G. Casas leg (CNAN-SC245), 4 juvs; • 16.XII.1998, 80 m, D. Estrada leg, 1 ♀ (CNAN-SC246) 1 ♂, 1 ♀; • 26.III.2000, 280 m, E. González-Santillán leg (CNAN-SC243); • 26.xi.2004, 80 m, A. Valdez leg (CNAN-SC269), 1 ♂; • 24.xi.2004, 13 m, A. Valdez leg (CNAN-SC271), 1 ♀; • 27.XII.1984, L.J. Vitt leg (CNAN-SC272) 22 juvs, 1 ♀; • Camino el Tejón, 18.VIII.2005, 19°29.84'N, 105°02.49'W, 86 m, J.L. Costelo, L.A. Gutierrez leg (CNAN-SC274), 2 ♀; • Playa Negritos, 15.V.2008, 19°31.76'N, 105°04.82'W, 31 m, S. Soriano, M. Vega, M. Hernández, C. Guzmán, R. Paredes leg (CNAN-SC247), 1 ♂, 1 ♀; • Playa Teopa, 19°24.06'N, 105°12.20'W, 7 m, E. González-Santillán leg (CNAN-SC244), 1 ♀.

**Figure 3.**
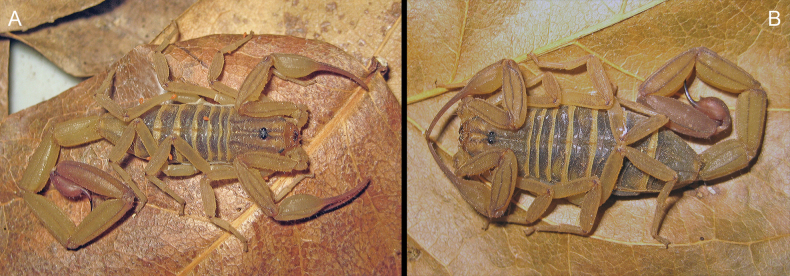
*Centruroideselegans* (Thorell, 1876). Live habitus of adult specimens from EBC**A** male **B** female.

##### Distribution.

Jalisco lowlands from Puerto Vallarta to Marabasco River, which is the natural border between Jalisco and Colima (González-Santillán et al. 2024).

##### Remarks.

This species is responsible for most of the acute envenomations reported in the coast of Jalisco (González-Santillán and Possani 2018). Our observations at the EBC indicated that is highly synanthropic, it is commonly found inside station buildings and dorms.

###### ﻿Family Vaejovidae Thorell, 1876

**Subfamily Syntropinae Kraepelin, 1905**

#### ﻿ Konetontli chamelaensis

Taxon classificationAnimaliaScorpionesButhidae

(Williams, 1986)

0F896628-FBB0-5381-9A41-53037F7682DE

Fig. 4A

##### Type material examined.

***Holotype*.** Mexico: • Jalisco, Municipio la Huerta: 1 ♂, allotype ♀, and seven ♂ topoparatypes, collected at Estación de Biología, Chamela, UNAM, 10–11.VII.1985, S. C. Williams leg, ultraviolet detection. Holotype depository: California Academy of Sciences, type no. 15744 (Williams 1986).

##### Other material.

Mexico: • Jalisco, Municipio de La Huerta: Estación de Biología Chamela, UNAM, 23.iv.1980, S.H. Bullok leg (CNAN-SC3850), 1 ♀; • 31.V.1990, N. Martínez leg (CNAN-SC3848), 1 ♀; • Vereda Verdin, 23.X.2001, E. González and J.L. Castelo leg (CNAN-SC3849), 1 ♂; • Rincón de Ixtlán, 19°329'N, 105°049'W, 24.X.2001, E. González leg (CNAN-SC3851), 1 ♀.

**Figure 4.**
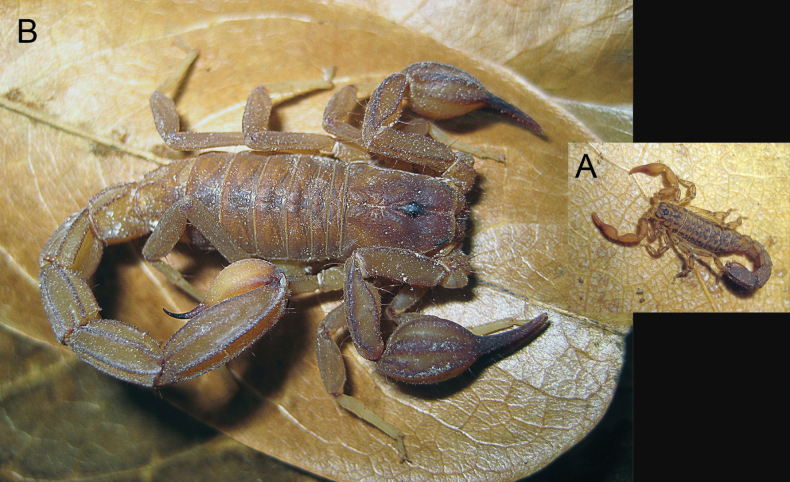
**A***Konetontlichamelaensis* (Williams, 1986) and **B***Thorelliusintrepidus* (Thorell, 1876). Live habitus of adult male specimens from EBC showing the size difference between the two species.

##### Distribution.

It has been reported only within the limits of the EBC.

##### Remarks.

*Konetontlichamelaensis* is an inhabitant of the leaf litter in the EBC and appears to be active on the surface during the rainy season (González-Santillán and Prendini 2015).

#### ﻿ Thorellius intrepidus

Taxon classificationAnimaliaScorpionesButhidae

(Thorell, 1876)

ACC55CB4-2BC2-53E6-AEE1-549F7E76CCB3

Fig. 4B

##### Type material.

***Holotype***. Mexico: • 1 ♀, deposited at Naturhistoriska Riksmuseet Stockholm, Sweden (not examined).

##### Other material.

Mexico: • Jalisco: Municipio la Huerta: Estación de Biología Chamela, 4.IV.1977, M. Varela-Juliá leg (CNAN-SC2244), 1 ♀; • 5.IX.1990, S.H. Bullock leg (CNAN-SC2920), 1 ♂; • 97 m, 19°29.875'N, 105°02.608'W, 31.VIII.2007, O. Francke, A. Valdez, H. Montaño, A. Ballesteros, C. Santibáñez leg (CNAN-SC2245), 3 ♂, 1 ♀; (CNAN-SC2617) ♀.

##### Distribution.

For a comprehensive compilation of records for this species refer to Chávez-Samayoa et al. (2024).

##### Remarks.

The ecology of this species is poorly known, despite being the largest species and being distributed in a wide range of altitudes, from sea level to almost 2000 m a.s.l. (Chávez-Samayoa et al. 2024), and its biological interactions and microhabitat are unknown. Like *K.chamelaensis*, its superficial activity occurs during the rainy season, but even then, it is not abundant.

#### ﻿ Mesomexovis caxcan sp. nov.

Taxon classificationAnimaliaScorpionesButhidae

FC8B973B-9B76-54AB-8D47-F74334E02C1D

https://zoobank.org/65880182-47DE-4CCC-AC15-1F2141902328

Figs 5 , 6 , 7 , 8 , 9 , 10 , 11 , 12 , 13 , 14 , 15 , 16 , Table 1

##### Type material.

***Holotype*.** Mexico: • Jalisco, Municipio La Huerta: ♂ (CNAN-T01869) and ***Allotype*** 1 ♀ (CNAN-T01870) Estación Biológica Chamela, IBUNAM, Sendero Calandria, 19°30.30'N, 105°2.30'W, 95 m, 4.X.2010, G. Montiel, R. Paredes, D. Barrales, and G. Contreras leg. ***Paratypes*.** Mexico: • Jalisco, Municipio La Huerta: Sendero Tejón, 19°30'N, 105°2.55'W, 63 m, 2.x.2010, G. Montiel, R. Paredes, D. Barrales, and G. Contreras leg, 2.X.2010 (CNAN-T01871), 8 ♂, 2 subadult 1 ♀.

##### Other material.

Mexico: • Jalisco, Municipio La Huerta, Estación de Biología Chamela, IBUNAM, Chamela-Cuixmala, 19°29.87'N, 105°2.61'W, 97 m, O. Francke, A. Valdéz, H. Montaño, A. Ballesteros, C. Santibáñez leg, 30.VIII.2007 (CNAN-SC02910), 3 ♂, 19 ♀, 9 juv., 39 2^nd^ instar juvs; • G. Casas leg, 3.VI.1975 (CNAN-SC2964), 1 ♂; (CNAN-SC2965), 2 ♂; E. Ramírez leg (CNAN-SC2963), 1 ♀; • D. Verduzco leg, 9.IX.2001 (CNAN-SC2967), 1 juv.; • D. Verduzco leg, 25.IX.2001 (CNAN-SC2971), 1 ♂; • M.A. Morales and A.M Corona leg, 12.XI.1998 (CNAN-SC02969), 1 ♀; • R. Ayala leg, 13.X.1993 (CNAN-SC2970), 1 ♂; • M. Córdova leg, X.2005 (CNAN-SC2972), 1 ♂; • A. B. López leg, 16.X.1994 (CNAN-SC2968), 4 ♂; • M. Guzmán, M. Hernández, R. Paredes, S. Solano, M. Vega leg, 13.V.2008 (CNAN-SC2953), 1 ♀, 1 juv.; • M. Ayala leg, 3.IX.1989 (CNAN-SC2952), 1 ♀; • A. Rodríguez leg, 20.VI.1994 (CNAN-SC2962), 1 ♂; • J. Guzmán leg, 28.VII.1989 (CNAN-SC2959), 1 ♂; • A. M. Corona leg, 29.III.1999 (CNAN-SC2958), 1 ♂; • A. López leg, 18.VI.1994 (CNAN-SC2961), 1 ♂, R. Ayala leg, 22.VII.1998 (CNAN-SC2960), 1 ♀; • N. Martí leg, 23.VIII.1990 (CNAN-SC2954), 1 ♀; • A. Rodríguez leg, 19.IX.1992 (CNAN-SC2956), 1 ♂; • E. Ramírez leg, 21.IX.1990 (CNAN-SC2955), 1 ♀; • E. González leg, 24.X.2001 (CNAN-SC2957), 1 ♀; • S.C. Williams leg, 12.VII.1985 (CNAN-SC02948), 4 ♀; • Sendero Tejón, E. González leg, 22.X.2001 (CNAN-SC2950), 6 ♀; • Pueblo Careyes, F. A. Noguera, A. Rodríguez leg, 20.VII.1999 (CNAN-SC2966), 1 ♀. • Bahía Chamela, L. Olguín, S. Reynaud, E. González leg, 21.X.2001 (CNAN-SC2949), 3 ♂, 3 ♀, 2 juvs; • Punta Pérula, orillas de estero, 19°35.46'N, 105°7.61'W, 20 m, 10.x.2017, C. Balderas, G. Balderas, T. Olamendi, H. Valencia, I. Gómez, E. González-Santillán leg (CNAN-SC4070), 14 ♂, 8 ♀.

##### Diagnosis.

*Mesomexoviscaxcan* sp. nov. is closely related to *M.occidentalis* (Hoffmann, 1931), *Mesomexovissubcristatus* (Pocock, 1898), and *Mesomexovisatenango* (Francke & González-Santillan, 2006) by having mesosomal tergites and ventral submedian carinae on metasomal segments III–IV granular. *Mesomexoviscaxcan* sp. nov. may be distinguished from *M.occidentalis* by having all carinae of pedipalp chelae outlined with dark pigmentation, costate, and weakly crenulate, instead of immaculate; except for dorsal retrolateral and ventral retrolateral carinae that are costate, all other carina are obsolete and smooth. *Mesomexoviscaxcan* sp. nov. differs from *M.subcristatus* by having ventral lateral carinae of metasomal segments I–IV granular and ventral submedian carinae of segments II–IV costate to granular, instead of costate to weakly crenulate and obsolete to weakly costate, respectively. *Mesomexoviscaxcan* sp. nov. differs from *M.atenango* by having dusky markings on carapace and mesosomal tergites, instead of diffused and weakly visible infuscation. Finally, *Mesomexoviscaxcan* sp. nov. differs from *M.atenango* and *M.subcristatus* by having three or four setae on the ventrolateral carinae of metasomal segment V instead of seven or eight, or ten or eleven, respectively.

##### Description.

The following description is based on the male type and male and female paratypes.

**Figure 5.**
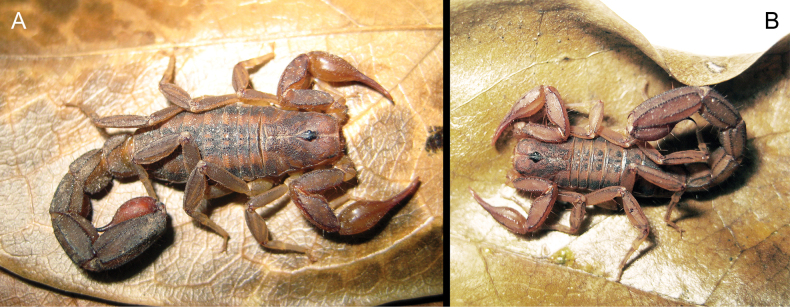
*Mesomexoviscaxcan* sp. nov. Live habitus of adult specimens from EBC**A** female **B** Male.

***Color and infuscation in alcohol***: base color yellowish to dark orange; carapace, tergites, and prolateral surface of legs femur, patella, tibia, and tarsi infuscate with dusky markings (Fig. 5). Pedipalp femur and patella with dorsal, prolateral, and retrolateral carinae outlined with diffuse infuscation. Pedipalp chelae with all carinae moderately infuscate. Pectines and genital operculum whitish. Dorsal median and dorsal submedian carinae in tergites I–VI outlined with intense infuscation. Sternite VII with base of setae on ventral submedian and ventral lateral carinae intensely infuscate, weakly converging in ventral submedian carina. Metasomal segments I–IV, ventral lateral and ventral submedian carinae, and metasomal segment V ventral lateral and ventral median carinae outlined with intense infuscation. Ventral surface of telson infuscate with two lateral and a median longitudinal broad band of dusky markings.

***Chelicerae***: manus dorsal surface smooth with one median macrosetae on subdistal margin, distal margin with costate carinae. Base of fixed finger with low crenulation. Serrula with 14 spines in distal half.

**Figure 6.**
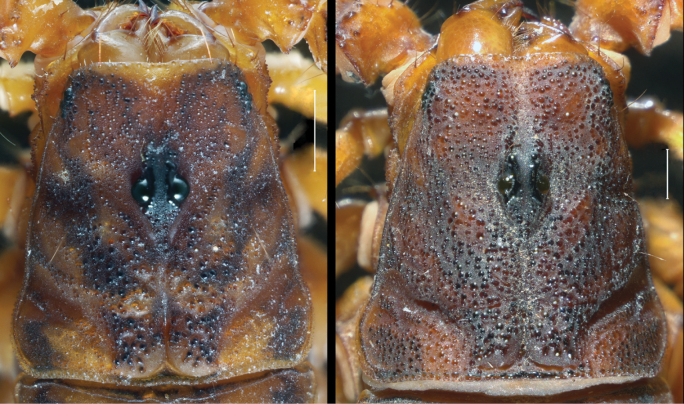
*Mesomexoviscaxcan* sp. nov. Carapace surface **A** adult female **B** adult male. Scale bar: 1 mm.

**Figure 7.**
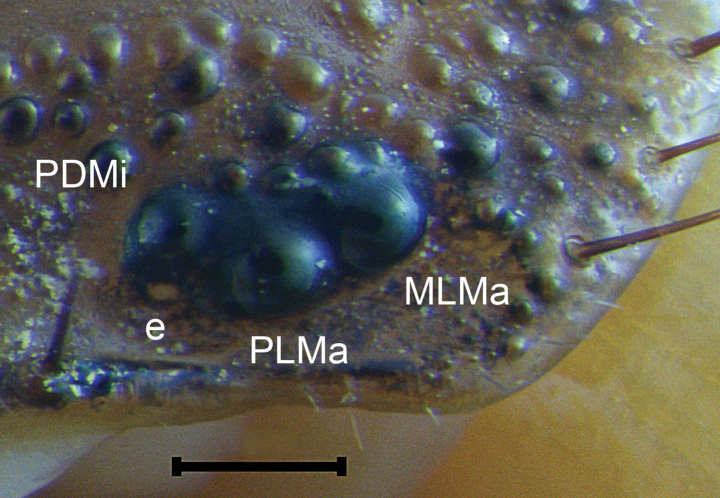
*Mesomexoviscaxcan* sp. nov. Lateral ocelli of an adult female from EBC. Scale bar: 1 mm. Abbreviations: e eyespot, MLMa mediolateral major ocellus, PDMi posterodorsal minor ocellus, PLMa posterolateral major ocellus.

***Carapace***: length 1.14/1.12× greater than posterior width (Table 1). Anterior margin emarginate, with a vestigial to obsolete median notch, with five pairs of major and a median macrosetae. Entire surface shagreened, with minute and moderately large granules over most of the surface (Fig. 6). Anteromedian, and posteromedial sulci moderate, posterolateral sulci deep, posterior transverse sulcus obsolete. Median ocular tubercle moderately raised, with superciliary carinae smooth, higher than median eyes, positioned on distal half of carapace. Median ocelli approximately double the size of lateral frontal pairs. Lateral ocelli of type 3A (Loria and Prendini 2014). Three lateral eyes, mediolateral major ocellus, posterolateral major ocellus similar size and posterolateral minor ocellus, reduced approximately in half, and with an eyespot ventrally (Fig. 7).

**Table 1.** Measurements (mm) of selected type material of *Mesomexoviscaxcan* sp. nov. deposited in the Colección Nacional de Arácnidos, IBUNAM.

**Catalog/Type**		**CNAN-T01869**	**CNAN-T01871**								**CNAN-SC2949**	
**Sex**		♂	♂	♂	♂	♂	♂	♂	♂	♂	♂	♂
Carapace	length	5	5	4.4	5.8	4.9	6.8	6	5.4	4.1	4	4.1
ant.	width	2.8	3	2.4	2.9	2.7	3.5	3.5	2.6	2.5	2.2	2.3
post.	width	4.4	4.6	3.6	4	4.2	5.9	6.1	4.6	3.8	3.8	3.5
Femur	length	3.9	4	3.1	4.2	4	6	5	3.9	3.3	3.3	3.2
	width	1.1	1.3	1	1.3	1.1	1.9	1.1	1.2	1	1	1
Patella	length	4.5	4.7	3.8	4.5	4.5	6.3	5.2	4.6	4	3.8	3.5
	width	1.4	1.5	1.2	1.6	1.5	2.1	1.6	1.8	1.2	1.3	1.2
Chela	length	7	7.5	5.6	7.2	6.5	10.5	8.6	7.2	6	4.6	5.5
Manus	width	2	2.3	1.7	2.3	2.1	3.5	2.5	2.3	1.8	1.6	1.6
	height	2	2.3	1.6	2.3	2	3	2.5	2.2	1.6	1.6	1.5
	length	4	4.5	3.1	4.2	3.5	5.5	4.6	4.7	3.8	2.1	3.2
Fixed finger	length	3	3	2.5	3	3	5	4	2.5	2.2	2.5	2.3
Mov. finger	length	4.2	4.5	3	4.1	4.1	6.5	5.2	4.2	3.6	3.5	3.5
Coxa II	length	2.1	2.1	1.7	2	2	3	2.4	2.1	2	1.7	1.5
Coxa IV	length	3.5	4	3.5	4.8	4.5	5.6	4.7	4.5	3.8	3.6	3.6
Sternum	length	1	1	0.8	1	1	1.2	1.1	0.9	0.8	0.6	0.5
	width	1.4	1.5	1.3	1.5	1.5	1.8	1.5	1.6	1.3	1.1	1.2
Mesosoma	length	9.9	10.4	8.8	11	10.6	14.4	13.5	10.9	7.6	8.4	8
Metasoma	length	19.1	18.7	15.2	19.3	19.2	27	22	19.8	15.8	14.9	15.4
Segment I	length	2.2	2.4	2	2.5	2.4	3.3	3	3	2	2	2
	width	3	3	2.4	3	2.8	3.9	3.5	3.1	2.3	2.4	2.4
	height	2.5	2.4	2	2.2	2.2	3.2	2.8	2.5	2	2	2
Segment II	length	3.1	3	2.3	3	3	4.3	3.5	3	2.5	2.2	2.4
	width	2.7	3	2.4	3	2.8	3.9	3.4	3.1	2.3	2.4	2.2
	height	2.2	2.5	2.1	2.3	2.3	3.2	2.8	2.5	2	2	2.1
Segment III	length	3.1	3.4	2.4	3.2	3.2	4.5	3.8	3.4	2.7	2.4	2.7
	width	2.7	3	2.4	2.9	2.8	3.8	3.3	3.1	2.3	2.3	2.2
	height	2.2	2.7	2.1	2.3	2.5	3.4	3	2.6	2	2	2.1
Segment IV	length	4	4.3	3.5	4.2	4.3	6.1	4.9	4.4	3.4	3.3	3.3
	width	2.8	3	2.4	2.9	2.8	3.6	3.3	3.1	2.3	2.3	2.1
	height	2.5	2.7	2.1	2.5	2.4	3.2	3	2.7	2.1	2.1	2.1
Segment V	length	6.7	5.6	5	6.4	6.3	8.8	6.8	6	5.2	5	5
	width	2.6	2.9	2.4	2.7	2.6	3.3	3	3	2.3	2.1	2.1
	height	2.3	2.4	2	2.3	2.3	2.8	2.3	2.5	2	2	2.1
Telson	length	5.5	6	4.5	5.8	5.5	7.5	6	5.6	4.6	4.2	4.4
Vesicle	length	3.5	4	2	3.8	3.5	5	3.4	4.6	3	2.7	2.9
	width	2	2	1.8	2	2	2.9	2.5	2.4	1.7	1.7	1.6
	height	1.6	1.7	1.4	1.8	1.7	2.1	2	2	1.3	1.3	1.3
Aculeus	length	2	2	1.5	2	2	2.5	2.4	2	1.6	1.5	1.5
Total	length	38	38.6	31.6	40.5	38.7	53.7	45.5	40	30.9	30.2	30.8
**Catalog /Type**		**CNAN-T1870**	**CNAN-SC2950**				**CNAN-SC2948**					
**Sex**		♀	♀	♀	♀	♀	♀	♀	♀	♀	♀	♀
Carapace	length	6.2	6.6	6.4	5.9	6.2	6.4	6.4	6	6.7	6.3	5.9
ant.	width	3.5	3.4	3.2	2.9	3.1	3.4	3.5	3.2	3.5	3.5	3.2
post.	width	5.2	6.3	5.4	5.1	5.6	5.6	5.5	5	6.2	5.9	5.4
Femur	length	4.7	5.3	4.6	4.5	4.8	4.8	5	4.3	5	4.8	4.5
	width	1.5	1.8	1.5	1.2	1.5	1.5	1.5	1.4	1.6	1.4	1.6
Patella	length	-	5.5	6	5.4	5.1	5.5	5.5	5.5	5	6	5.6
	width	1.7	2	1.6	1.5	1.9	1.8	2	1.8	2	1.7	2
Chela	length	8.3	9.3	8.4	7.2	8.2	8.5	8.6	7.9	9.5	8.7	7.7
Manus	width	2.5	2.8	2.3	2.1	2.5	2.5	2.5	2.1	2.8	2.5	2.5
	height	2.5	2.9	2	2.5	2.4	2.6	2.4	2.3	3.2	2.6	2.5
	length	4.3	4.8	4.4	3.7	4.2	4.5	4.6	4.2	4.5	4.7	3.7
Fixed finger	length	4	4.5	4	3.5	4	4	4	3.7	5	4	4
Mov. finger	length	5.2	5.3	5	4.8	5.3	5.4	5	4.8	5.5	5.5	4.8
Coxa II	length	2.4	2.7	2.7	2.6	2.5	2.5	2.5	2.3	2	2.5	2.2
Coxa IV	length	5.1	6.3	5.7	5	5.5	5.4	5.2	4.8	6	5.5	5.2
Sternum	length	1	1	1	0.8	1	1	1	1	1.1	1	0.9
	width	1.7	1.7	1.7	1.5	1.5	2	2	1.5	1.8	1.8	1.6
Mesosoma	length	15	17.3	14.3	13.2	16.5	15.1	13.9	13.7	14	12.4	13.6
Metasoma	length	21	22.5	21.3	20.7	22.2	21.7	22.2	20.8	23.7	21.6	19.9
Segment I	length	2.7	3.4	3.2	3	3	3	3.2	3.2	3.3	3	2.8
	width	3.6	4.1	3.4	3.3	3.7	3.6	3.7	3.5	4.1	3.7	3.5
	height	2.8	2.5	2.8	2.7	2.9	2.7	2.8	2.8	3.2	3	2.8
Segment II	length	3.3	3.6	3.3	3.3	3.6	3.5	3.3	3.3	3.8	3.2	3.1
	width	3.5	4	3.4	3.2	3.5	3.6	3.7	3.2	4	3.6	3.5
	height	2.9	2.5	2.8	2.7	2.9	2.7	2.8	2.8	3.2	2.9	2.8
Segment III	length	3.6	3.8	3.4	3.4	3.8	3.7	3.6	3.4	3.9	3.5	3.5
	width	3.5	3.9	3.4	3.1	3.3	3.5	3.5	3.3	4	3.5	3.5
	height	3	2.6	3	2.8	2.9	2.8	2.7	2.8	3.3	2.9	2.8
Segment IV	length	4.7	4.5	4.6	4.2	4.8	4.5	5	4.5	5.2	4.9	4
	width	3.5	3.8	3.4	3.1	3.3	3.5	3.5	3.3	3.9	3.5	3.1
	height	3.1	3	3	2.8	3	3	2.8	2.8	3.4	3	3
Segment V	length	6.7	7.2	6.8	6.8	7	7	7.1	6.4	7.5	7	6.5
	width	3.5	3.5	3.2	3.1	3.3	3.5	3.4	3.4	3.7	3.4	3
	height	2.9	2.8	2.4	2.7	2.5	2.5	2.8	2.8	2.9	3	3
Telson	length	6	7	6	6	6.1	6.5	6.6	6	7.2	6.6	6.2
Vesicle	length	4	4.3	4	4	4	4.5	4.4	4	5	4.4	4.2
	width	3	3.3	2.8	2.7	2.6	2.8	3	2.8	3.5	2.8	2.7
	height	2.2	2.5	2.1	2	2.3	2.3	2.4	2.2	2.8	2.2	2
Aculeus	length	2	2.7	2	2	2	2	2.2	2	2.2	2.2	2
Total	length	46.1	51.2	45.9	43.8	48.8	47.5	47	44.4	49.5	45	43.5

**Figure 8.**
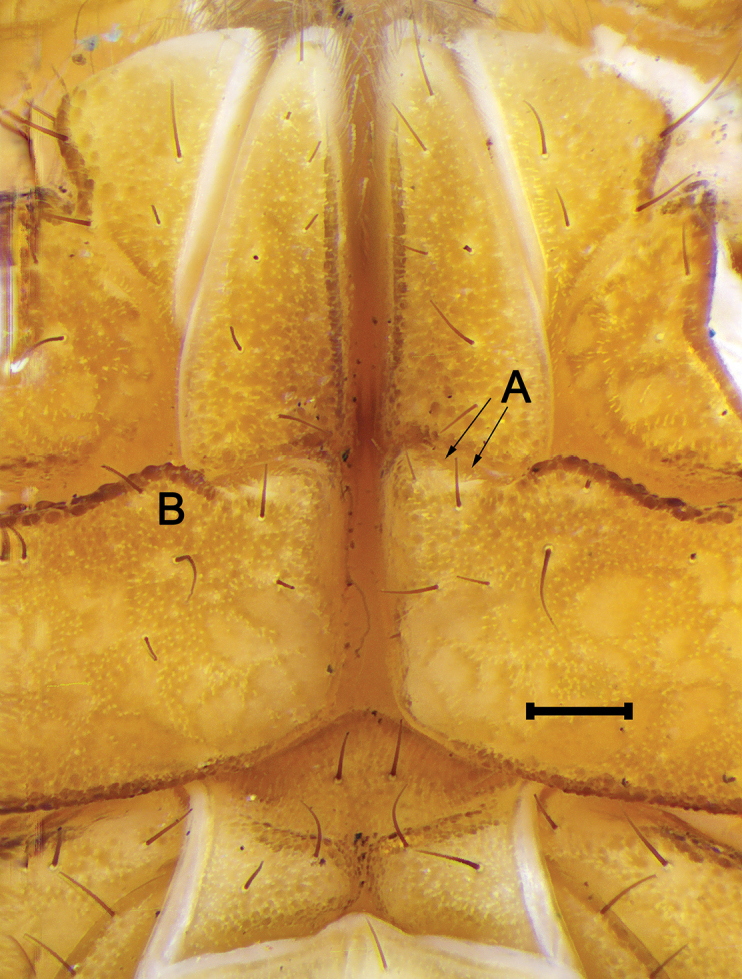
*Mesomexoviscaxcan* sp. nov. Coxae I and II of adult male from EBC**A** slit-like structures **B** strong and crenulated protuberance.

***Coxosternal region***: sternum subequilateral pentagonal; width 1.63/1.74× greater than length (Table 1); with four pairs of setae, one distal, two median pairs, and one pair on the lobes, median notch deep. Anterior margin of coxae II with two oblique slit-like structures (Fig. 8A), adjacent to a strong and crenulate protuberance (Fig. 8B). Coxal endite II proximal median margin with a deep depression. Coxa IV 2.03/2.20× longer than coxa II (Table 1).

**Figure 9.**
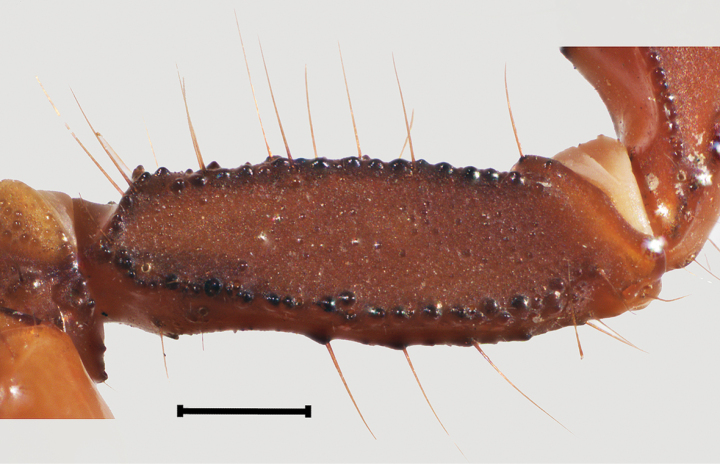
*Mesomexoviscaxcan* sp. nov. Femur dorsal surface of adult male from EBC. Scale bar: 1 mm.

**Figure 10.**
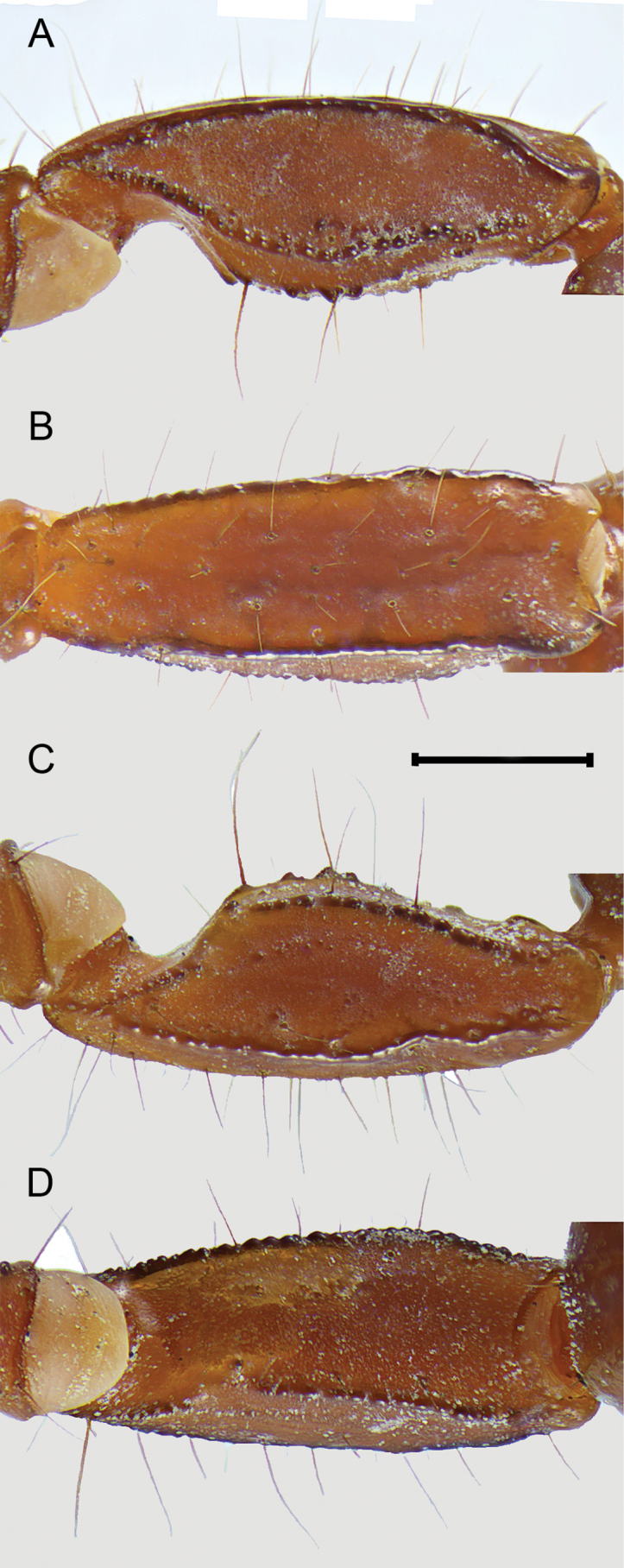
*Mesomexoviscaxcan* sp. nov. Patella of an adult male from EBC**A** dorsal surface **B** retrolateral surface **C** ventral surface **D** prolateral surface. Scale bar: 1 mm.

**Figure 11.**
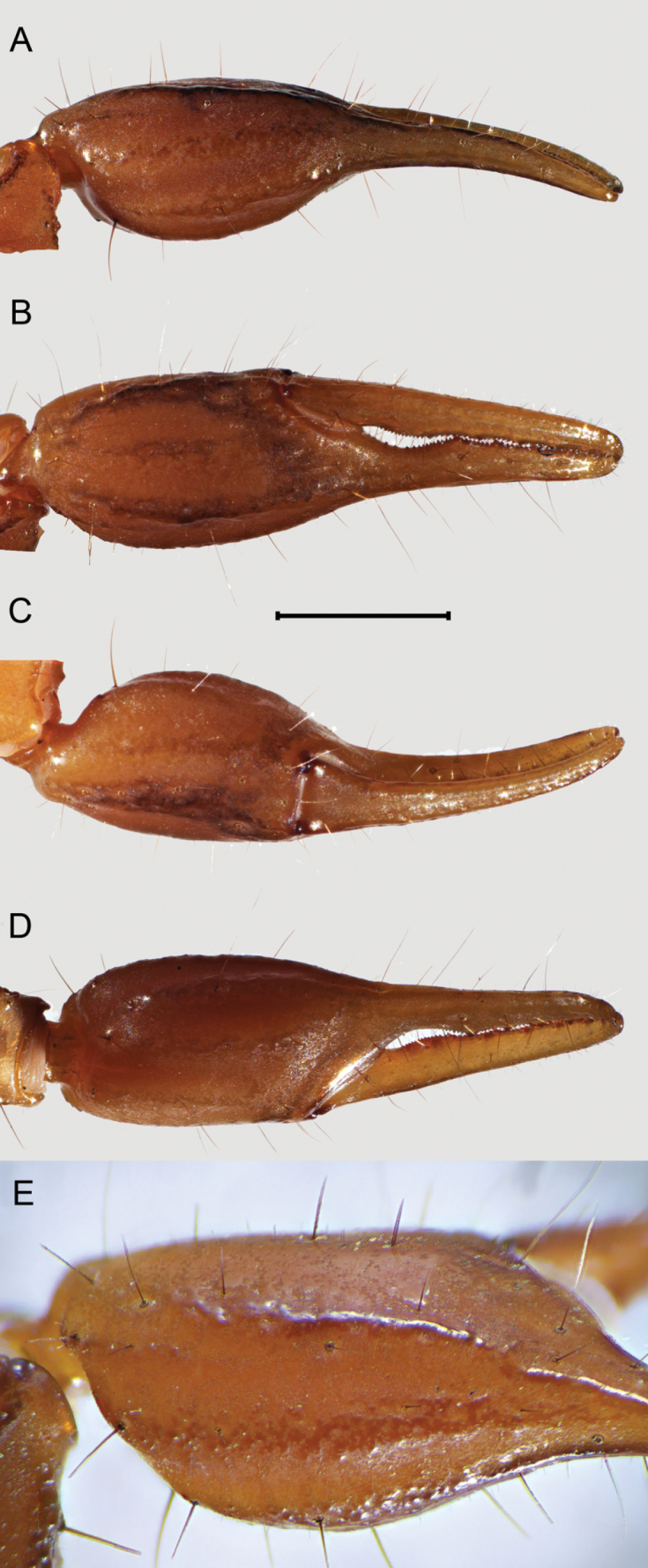
*Mesomexoviscaxcan* sp. nov. Pedipalp chelae of an adult male from EBC**A** dorsal surface **B** retrolateral surface **C** ventral surface **D** prolateral surface **E** magnified dorsal surface (2×) to show costate crenulate carinae. Scale bar: 5 mm.

***Pedipalps***: femur and intercarinal surfaces matte, dorsal and ventral surfaces with scattered minute to moderate median granules (Fig. 9). Dorsal prolateral, dorsal retrolateral, and ventral prolateral carinae granular; retrolateral dorso-submedian carina obsolete, smooth with four major macrosetae, distal one reduced; prolateral ventro-submedian carina vestigial with an enlarged proximal tubercle positioned medially with a row of four or five moderate to large rounded granules/ subdistal, median and distal granules with a macroseta at the base, slanting gently towards median portion of dorsal prolateral carinae; prolateral ventral carina vestigial, expressed by a proximal tubercle with a macroseta at base and two enlarged rounded granules with a macroseta at base; intercarinal surface between prolateral ventral and ventral prolateral with three macrosetae; ventral median and retrolateral ventral carinae vestigial expressed by scattered serrated granules; ventral retro-submedian carinae expressed by irregular granules, present on proximal half. Patella 1.27/1.21× wider than femur (Table 1). Intercarinal surfaces matte (Fig. 10). Dorsal prolateral and ventral prolateral carinae complete, granular; dorsal retrolateral and ventral retro-submedian carinae complete, crenulated to smooth; ventral median carina vestigial expressed as a short proximal row of granules; retrolateral dorso-submedian and retrolateral median carinae obsolete, expressed by bands of infuscation and a weak median costa; prolateral process well-developed; prolateral median carina expressed as a row of five to six coarse subserrated granules, median one with a macroseta, the row slanting gently at the level of trichobothrium *d_2_* towards dorsal prolateral carinae, followed by a gap of smooth surface and ending on one distal major macrosetae, positioned on the distal fifth of dorsal prolateral carinae; prolateral ventral carina obsolete expressed by a row of three major macrosetae and two intersperse minor macrosetae, proximal major macrosetae on the top of a low granule. Chela 1.52/1.51× longer than patella, 1.72/1.76× longer than femur (Table 1). Manus relatively slender (Fig. 11), 1.42/1.35× wider than patella, 1.80/1.64× wider than femur. Intercarinal surface smooth with a weak reticular infuscation. Dorsal median, dorsal retrolateral, dorsal prolateral, and prolateral median carinae complete costate finely granular (Fig. 11E); ventral retrolateral carina incomplete moderately costate, ending by the level of trichobothrium *Est*; ventral median carina weak, crenulate; ventral prolateral carina moderately costate. Trichobothrium *Db* positioned on dorsal retrolateral carina, subbasal on chelae; *Dt* positioned close to mid manus. Fixed and movable fingers dentate margins emarginate, fixed finger proximal notch strong and median lobe moderate, movable finger proximal notch weak and median lobe moderate, large gap created when fingers closed, fingers fit unevenly (♂), or proximal notch and median lobe of fixed finger and movable fingers gap created when fingers closed moderated (♀). Fixed finger median denticle row comprising six denticle subrows flanked by six prolateral and six retrolateral subserrate, subpaired denticles, proximal retrolateral denticle positioned by proximal third of finger; movable finger median row comprising seven denticle subrows, flanked by seven prolateral and seven retrolateral denticles, proximal retrolateral denticle positioned by proximal third of finger. Trichobothria *ib* and *it* positioned submedian on fixed finger, *ib* positioned adjacent to sixth prolateral denticle and *it* between fifth and sixth prolateral denticle (♂). Trichobothrial pattern Type C, orthobothriotaxic.

**Figure 12.**
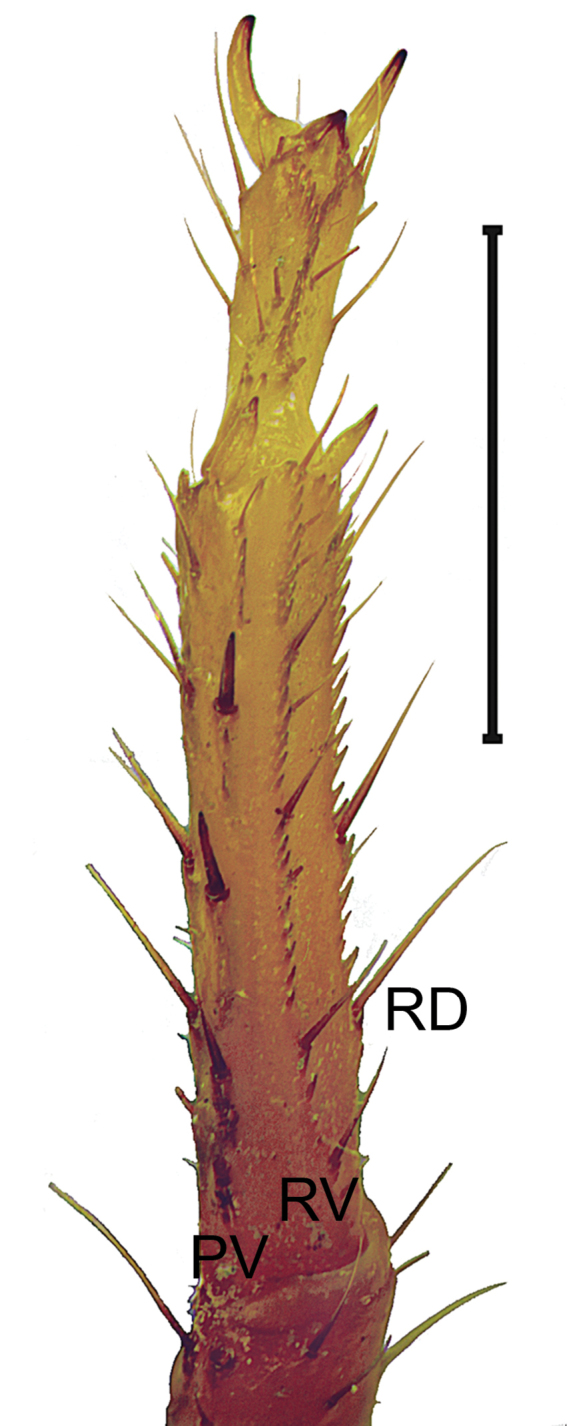
*Mesomexoviscaxcan* sp. nov. Dextral leg III, basitarsus and telotarsus, ventral aspect of an adult male from EBC. Abbreviations: PV, prolateral ventral. RV, retrolateral ventral. RD, retrolateral dorsal. Scale bar: 2 mm.

***Legs***: Basitarsi I–III prolateral ventral, retrolateral ventral and retrolateral dorsal carinae with complete rows of spinules, basitarsi IV prolateral ventral and retrolateral ventral carinae devoid of spinules, retrolateral dorsal carina with scattered spinules. Macrosetal counts on carinae of legs I–IV, respectively: Prolateral ventral, 3:4:5:5, proximal three in leg III and IV stouts (Fig. 12); retrolateral ventral, 4:7:8:8; retrolateral median, absent; retrolateral dorsal, 2-3:2-3:2-3:2-3; dorsal, 2-3:2-3:2-3:2-3; retrolateral dorsal and dorsal series arranged in two independent rows. Telotarsi I–IV with two to three pairs of ventrodistal spinules (Fig. 12); macrosetal counts on legs I–IV proventral/retroventral: 0/0:1/1:1/1:2/2 unpaired, retroventral distal, proventral proximal.

***Genital operculum***: Wider than long, with three pairs of macrosetae. Sclerites free on longitudinal edges moving independently but unable to open more than 45°. Genital papillae protruding posteriorly (♂) or sclerites fuse longitudinal (♀).

**Figure 13.**
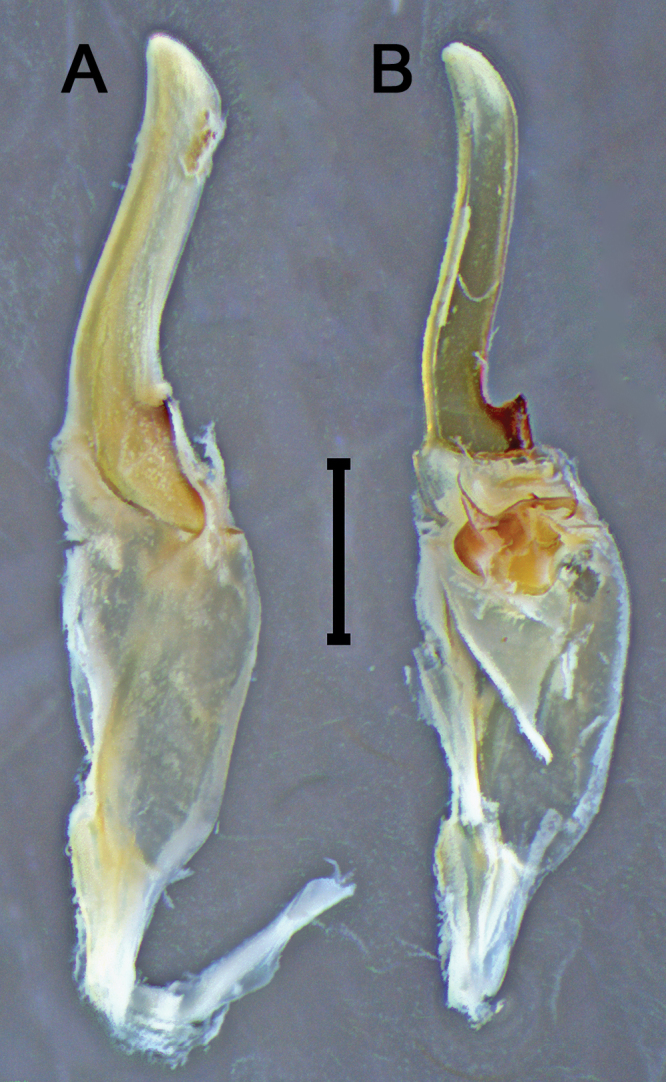
*Mesomexoviscaxcan* sp. nov. Dextral hemispermatophore in toto **A** lateral aspect **B** contra-lateral aspect with partial dissected hemi-mating plug. Scale bar: 1 mm.

**Figure 14.**
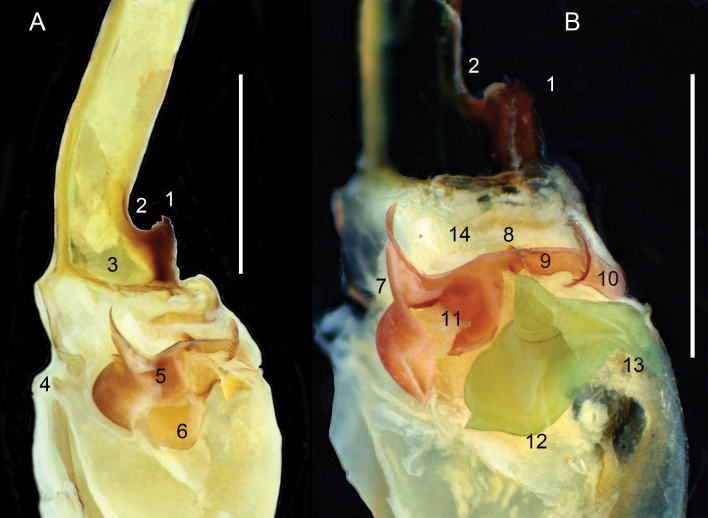
*Mesomexoviscaxcan* sp. nov. Dextral hemispermatophore, capsule zoom with hemi-mating plug cleared showing microstructures **A** contra-lateral aspect **B** zoom of contra-lateral aspect. Numbers: 1. Laminar hook. 2. Laminar base notch. 3. Contralateral though. 4. Truncal flexure. 5. Hemi-mating plug (Basal carina). 6. Subex. 7. Basal piece of hemi-mating plug. 8. Lateral spine. 9. Distal barb. 10. Distal retro-barbed margin. 11. Spatulated projection. 12. Pouch-like invagination. 13. Sperm duct. 14. Terminal membrane. Scale bars: 0.5 mm.

**Figure 15.**
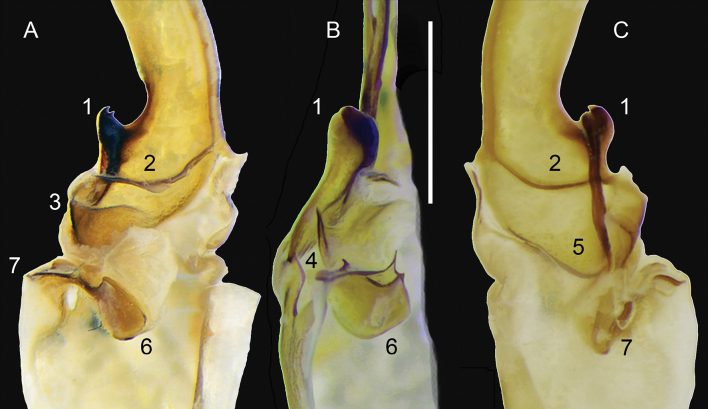
*Mesomexoviscaxcan* sp. nov. Sinistral hemispermatophore with hemi-mating plug removed showing microstructures **A** contra-lateral aspect **B** anterior aspect **C** lateral aspect. Numbers: 1. Laminar hook. 2. Contralateral though. 3. Tectum. 4. Capsular foramen. 5. Lateral through. 6. Pouch-like invagination. 7. Sperm duct. Scale bars: 1 mm.

***Hemispermatophore***: Lamina 1.3× longer than stem (Fig. 13). Laminar hook positioned on the apex of distal carina (Figs 14A–1, B-1, 15A-1, C-1). Hook bicuspid, short and low, separated from the anterior margin of the laminar base, forming a shallow notch (Figs 14A–1, 15A–1, C-1). Contralateral through strongly sclerotized (Fig. 15A–2, C-2). Lateral through margin short, wide, tapering, and curving proximally, moderately removed from contra-lateral through (Fig. 15C–5). Architecture of the capsule conforms to the three-fold bauplan (Monod et al. 2017). Hemi-mating plug developed from the basal carina (14A-5, 6), distal barb margin with 14 elongated spinules (Fig. 14B–10). Basal carina posterior surface or basal plate (González-Santillán and Prendini 2013) rounded with two extensions forming a double wing-like structure and with a second spinoid projection raising towards the opposite side of the wing-like structure (Fig. 14B–7). Distal barb with one strongly sclerotized spine localized on the lateral surface pointing backwards (Fig. 14B–8). Base of the distal barb with a spatulated strongly sclerotized projection on contralateral surface (Fig. 14B–11). Distal barb 1.5× longer than the basal plate. Subex with two structures (Figs 14A–6, B-12, 15A-6, B6), the pouch-like invagination, rounded with two sharp projections situated on the posterior aspect (Figs 14B–12, 15B–6) and connects to the anterior aspect with the sperm duct, a spatulated projection adjacent to the capsular foramen (Figs 14B–13, 15A–7, B-4). Terminal membrane, thick, tightly connects the tectum (Fig. 15A–3), basal carina, and subex (Fig. 14B–14). Truncal flexure prominent (Fig. 14A–4). Measurements: lamina, 3.22; trunk, 2.46; dorsal trough/laminar hooks, 0.81; ventral trough/laminar hook, 0.34; distal barb, 1.26; basal plate, 1.96; pedicel 1.17.

**Figure 16.**
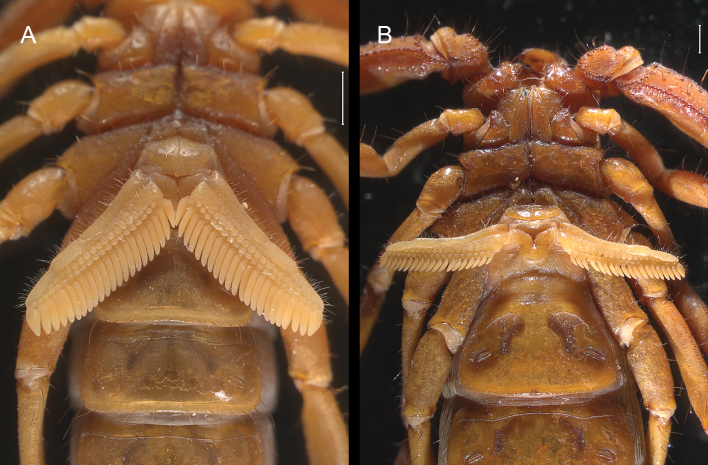
*Mesomexoviscaxcan* sp. nov. Ventral aspect of coxosternal region, pectines, and mesosomal segments I–III **A** male **B** female. Scale bar: 1 mm.

***Pectines***: longer than distal edge of coxa IV (♂) or at the level of distal edge of coxa IV (♀). Basal piece with median notch deep (Fig. 16). Marginal lamella consists of three sclerites, medial lamella proximally fuses distally progressively forming conspicuous 14 or 15 (♂), or 12 or 13 (♀) beads. Fulcra with 19–21 (♂), 17–20 (♀) beads. Pectinal tooth count 20–22 (♂) or 18–21 (♀).

***Tergites***: pretergites and postergites matte proximally, postergites distally shagreened. Tergites I–VI dorsal median and ventral submedian carinae vestigial, expressed by few moderate or minute distal granules (♂), or granular (♀) (Fig. 5). Tergite VII with intercarinal surfaces shagreened; dorsal median carina costate-granular expressed on proximal half of the postergite; dorsal submedian vestigial expressed as a short, oblique, proximal row of granules; dorsal lateral and lateral supramedian granular, merging proximally creating a semi ring of strong serrated granulation.

**Figure 17.**
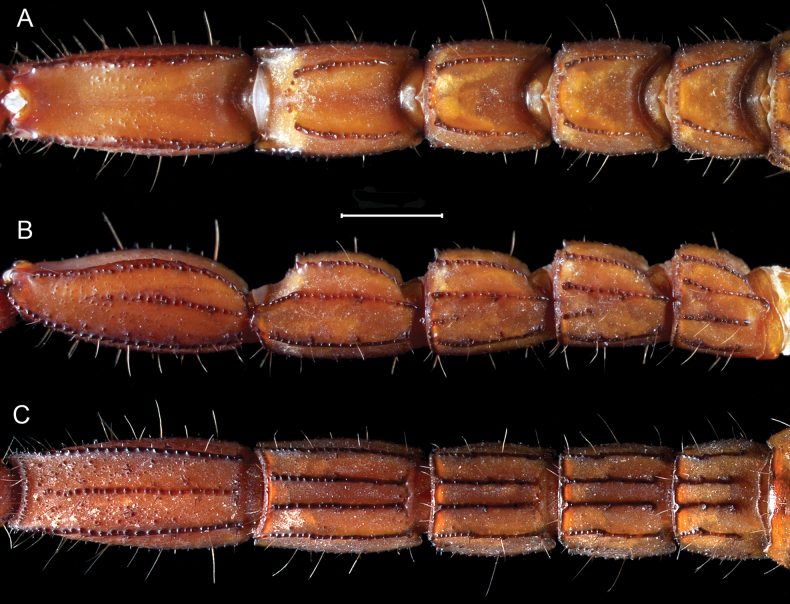
*Mesomexoviscaxcan* sp. nov. Metasomal segments I–V **A** dorsal aspect **B** lateral aspect **C** ventral aspect. Scale bar: 2 mm.

***Sternites***: I–VI smooth, matte in (♂), in ♀ finely punctuated, and lustrous (Fig. 17). Sternite V distal margin with a triangular, whitish glandular area (♂) or (♀) homogeneously colored. Sternite VII matte laterally and punctuated smooth ventrally; ventral lateral carinae partial, granular, expressed on median third; ventral submedian obsolete expressed by three pairs of macrosetae. Spiracle elongated, slit-like, ~ 3× longer than wide.

**Figure 18.**
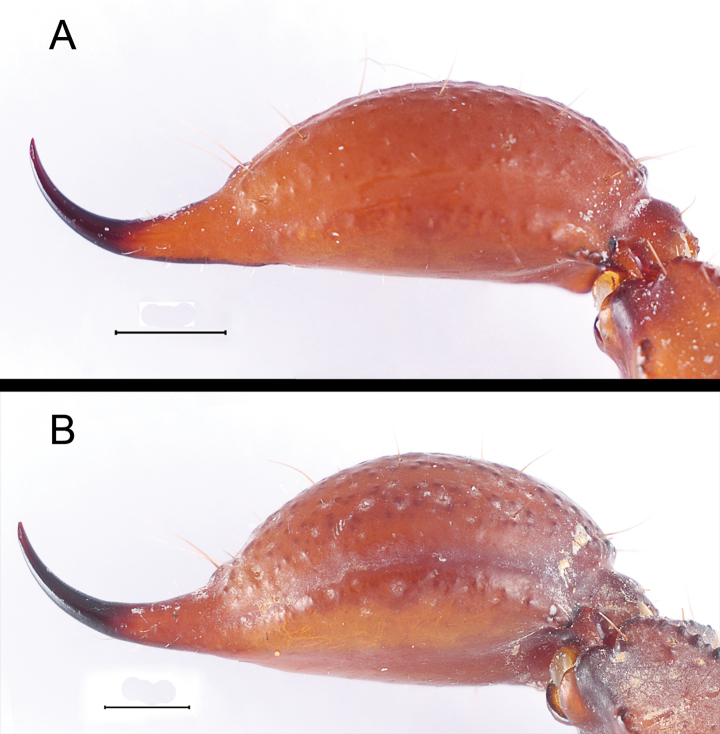
*Mesomexoviscaxcan* sp. nov. Telson, lateral aspect **A** male **B** female Scale bar: 1 mm.

***Metasoma***: 1.82/1.49× longer than mesosoma. Segments I–V, respectively 0.84, 1.03/0.95, 1.13/1.03, 1.50/1.34, 2.30/2.06× longer than wider; V, 1.30/1.16× wider than telson vesicle. Segments I–V, dorsal and lateral intercarinal surface with fields of scatter granules medially (Fig. 17), ventral surface of metasomal segment I and III smooth with few small granules, IV and V smooth with few fields of granules medially more so on V; dorsal lateral and lateral median carinae on I–IV, moderately serrated; lateral median carina on IV distal terminus expanded or flared laterally forming a triangular projection (Fig. 17A, B); dorsal lateral carina on V proximally third strongly serrated, becoming weaker and crenulated with irregular clusters of crenulations medially, moniliform distally; lateral inframedian carina on I complete serrated; on II incomplete serrated, becoming weaker proximally, present in distal third; on III incomplete serrated, present in distal fourth; on IV and V absent (Fig. 17B); ventral lateral carina on I–V strongly serrate (Fig. 17C); ventral submedian carina on I costate and smooth, on II costate and smooth on with distal end crenulate, on III weakly costate and smooth on two proximal thirds distal third crenulate, on IV proximal half smooth, distal half serrated, on V absent; ventral median carina on V strongly serrate (Fig. 18C). Macrosetal counts on carinae of segments I–V, respectively: Dorsal lateral, 1:3:3:3:5; lateral median, 2:3:3:4:5; lateral inframedian, 2:1:1:0:0; ventral lateral, 3:4:4:4-6:10, setae on V positioned laterally to the carinae instead on the carina; ventral submedian, 3:3:3:3-5:6; ventral sublateral, 2. Accessories macrosetae absent.

***Telson***: vesicle 1.70/1.46× longer than wide; 1.83/2.01× longer than aculeus; elongated, dorsal surface smooth to moderately granular (♂) or comparatively more globose and more granular (♀) (Fig. 18A, B). Subaculear tubercle moderately broad and low. Microserration on laterobasal aculeus 3–5 (♂), or 2–3 (♀) (Fet et al. 2006).

##### Distribution and ecology.

*Mesomexoviscaxcan* sp. nov. is distributed along Jalisco’s littoral and Colima. Inhabits tropical dry forest, including tropical deciduous forest and tropical broadleaf forest. This species was identified as a lapidicolous (González-Santillán 2004), although we found that the prolateral ventral carina on legs III and IV are armed with three, proximal, stout macrosetae (Fig. 12), which are characteristic of fossorial scorpions. At the EBC, the species is commonly found under rocks and rotten logs, although during recent fieldwork we observed females using abandoned burrows in a sit-and-wait foraging strategy. Males exhibit increased surface activity during the rainy season and thus are easily spotted during the night using ultraviolet detection.

##### Remarks.

López-Granados (2019) conducted a phylogenetic analysis on specimens from Chamela, Jalisco, and Coquimatlán, Colima that formed a monophyletic group with two synapomorphies, body color brownish and ventral surface of metasomal segment V with setae basis pigmented and areolas unpigmented. Granados-López observed that the number of setae on the carinae was lower in this species than in others. For instance, *M.caxcan* sp. nov. had only five setae on the fifth segment carina, whereas other species had as many as 12 (López-Granados 2019). The author diagnosed the clade with the following features: the carina on the pedipalp manus and the first three ventral segments of the metasoma tended to be costate to crenulate. Our observations confirm these features in all examined Chamela specimens. López-Granados (2019) identified seven clades that potentially represent undescribed species, among which *M.caxcan* sp. nov. is the closest to the outgroup. Remarkably, all clades maintain a constant pattern with their distribution. *Mesomexoviscaxcan* sp. nov. represents the first divergent clade and occupies the northern limit of distribution range of the species complex. The remaining clades follow a laddered arrangement from the more ancient to the more recently divergent clade following a distribution north-south on the Pacific lowland biogeographic province identified by Morrone et al. (2017). González-Santillán et al. (2024) concluded that genetically, the specimens from Jalisco and Colima are not conspecific with the specimens identified as *M.occidentalis* (González-Santillán and Prendini 2015), confirming the earlier observation of Sissom (1989). These observations justify the description of *M.caxcan* sp. nov. from a morphological, biogeographical, and genetic perspective.

##### Etymology.

The species name *caxcan* is derived from the name of one of the original nomadic groups of Chichimeca peoples that occupied the arid land of northern Mexico. The name is a noun in apposition.

### ﻿Updated key to the identification of scorpions of the Estación de Biología Chamela

**Table:** 

1	The ventral surface of the pedipalp patella without trichobothrial series *v_1_*–*v_3_*, sternum subtriangular	**Buthidae 2**
–	The ventral surface of the pedipalp patella with trichobothrial series *v_1_*–*v_3_*, sternum pentagonal (Fig. 9C)	**Vaejovidae 3**
2	Pectinal tooth count 22–26 (♂) or 20–25 (♀), anterior margin of the pectinal basal piece in females V-shaped with a shallow median depression, telson base color reddish	***Centruroideselegans* (Thorell, 1876)**
–	Pectinal tooth count 17–20 (♂) or 15–18 (♀), anterior margin of the pectinal basal piece in females straight with a deep median depression, telson base color yellowish	***Centruroideschamela* Ponce-Saavedra & Francke, 2011**
3	Telson vesicle with spiniform subaculear tubercle (Fig. 4A), pedipalp chelae carina obsolete	***Konetontlichamelaensis* (Williams, 1986)**
–	Vesicle without subaculear tubercle (Figs 4B, 5), pedipalp chelae carina granular to costate	**(4)**
4	Pedipalp chela incrassate with broad, granular carinae (Fig. 4B); ventral and ventral submedian carinae of metasomal segments I–IV immaculate	***Thorelliusintrepidus* (Thorell, 1876)**
–	Pedipalp chela comparatively slender with costated to obsolete carinae (Fig. 5); ventral and ventral submedian carinae of metasomal segments I–IV outlined with infuscation	***Mesomexoviscaxcan* sp. nov.**

## ﻿Discussion

In this contribution, we formally described *Mesomexoviscaxcan* sp. nov. as part of a faunal assemblage of five scorpion species comprising two *Centruroides* species of medical importance (González-Santillán and Possani 2018) and three vaejovids belonging to divergent monophyletic and ecomorphological groups (González-Santillán and Prendini 2013, 2015). Little knowledge about the niche use and phenology of these species has been generated. *Centruroideselegans* is an errant species like many buthids around the world (McReynolds 2008; Stevenson et al. 2012; Lira et al. 2018), whereas *C.chamela* is an arboreal species (González-Santillán 2004; Ponce-Saavedra and Francke 2011). *Thorelliusintrepidus* thrives in tropical habitats with altitudinal limits of ~ 1800–2000 m (Chávez-Samayoa et al. 2024; González-Santillán et al. 2024). Although presumed a lithophilous species, it may exhibit a similar ecomorphotype as *M.caxcan* sp. nov., but more field evidence is needed to clarify this. Finally, *K.chamelaensis* is a small (11–13 mm) humicolous species whose superficial activity appears to be restricted to the rainy season (Williams 1986; González-Santillán and Prendini 2015). A similar pattern was observed in others humicolous species that inhabit South American dry forests, such as *Ananteris* spp. (Lira et al. 2018). Overall, *M.caxcan* sp. nov. is difficult to assign to a known ecomorphotype. On the one hand, it seems to be a lapidicolous species, and at the same time, it appears to be a fossorial species, as observed in the field. This seems to be the pattern found in dry forest areas: in the Brazilian Caatinga dry forest, some mid-sized species such as *Jaguajirrochae* (Borelli, 1910) and *Tityusstigmurus* (Thorell, 1876) are also difficult to attribute to any ecomorphotype (Lira et al. 2018). However, studies on the use of microhabitats by scorpions in Chamela, as well as other dry forest areas, need to be conducted to test these hypotheses.

### ﻿On the hemispermatophore of *Mesomexoviscaxcan* sp. nov.

Monod et al. (2017) identified five structural elements: the distal carina, tectum, subex, terminal membrane, and basal carina, which constitute the capsular area of the hemispermatophore of scorpions. They delimited these structures in most families of the Order Scorpiones, including the family Vaejovidae. Although the authors included Smeringurinae and Vaejovinae, they omitted members of the subfamily Syntropinae. Here, we describe for the first time the main capsular pattern in the subfamily Syntropinae illustrated by *M.caxcan* sp. nov. We found a sharp difference in the limits of the subex and the basal carina. Monod et al. (2017: figs 18, 19) illustrated the basal carina as a continuous extension of the sperm duct, at least for members of the subfamilies Smeringurinae and Vaejovinae. Our comparative study indicates that the basal carina in *M.caxcan* sp. nov. is not directly connected to the subex (Figs 6B, 14A–5). Thus, the subex comprises the pouch-like invagination and a structure that Stockwell (1989) identified as sperm duct, which is a sclerotized spatulated structure that putatively creates an auxiliary sclerotized piece of the sperm duct sensu Monod et al. (2017) most likely involucrate during the sperm transference. This pattern may be expressed in the subfamilies Smeringurinae and Vaejovinae but warrants future studies.

A second line of evidence supporting the idea that the sperm duct is part of the subex is that, during dissection of the hemi-mating plug, the hemi-mating plug detaches once the engulfing membrane is removed, leaving the sperm duct and the subex, or pouch-like invagination, connected (Figs 7B–6, 15A–6). It is evident that there is a separation between the basal carina (Fig. 14B) and the subex, the former sits on the latter, which is consistent functionally. The robust sclerotization of the subex may form a sturdy base that securely supports the sperm duct, ensuring it functions effectively. The more “external” position of the mating plug made by the basal carina may facilitate the extrusion from the capsular area and introduction into the female’s genital atrium. In summary, our study generates a better interpretation of the morphology and functionality of the capsular area of the hemispermatophore that may aim to understand the evolution and systematics of the North American family Vaejovidae.

## ﻿Conclusions

Our knowledge of the scorpion assemblage known to inhabit the EBC is almost limited solely to taxonomic diversity. Future research should focus on discovering the ecological diversity of these scorpion assemblages to understand the role of these species in the ecosystem. Restoration, conservation, and preservation programs should be harnessed with a clear ecological and biological picture of the function of the members of the ecosystem; thus, our taxonomic contribution describing *M.caxcan* sp. nov. might help build a more robust framework for that purpose. Furthermore, we suggest that this information could result in shedding some light on the biology and autecology of medically relevant scorpion species, still a gruesome public health problem in Mexico (González-Santillán and Possani 2018), to prevent, treat, and abate the number of accidents of intoxication by scorpion stings.

## Supplementary Material

XML Treatment for Centruroides chamela

XML Treatment for Centruroides elegans

XML Treatment for Konetontli chamelaensis

XML Treatment for Thorellius intrepidus

XML Treatment for Mesomexovis caxcan

## ﻿Additional information

### Author ORCIDs

André Felipe de Araujo Lira https://orcid.org/0000-0002-8443-4126

Edmundo González-Santillán https://orcid.org/0000-0003-1340-068X

### Data availability

All of the data that support the findings of this study are available in the main text.
